# ADAMTS-1 Is Found in the Nuclei of Normal and Tumoral Breast Cells

**DOI:** 10.1371/journal.pone.0165061

**Published:** 2016-10-20

**Authors:** Suély V. Silva, Maíra A. Lima, Nathalie Cella, Ruy G. Jaeger, Vanessa M Freitas

**Affiliations:** Department of Cell and Developmental Biology, Biomedical Sciences Institute, University of São Paulo, São Paulo, Brazil; CHA University, REPUBLIC OF KOREA

## Abstract

Proteins secreted in the extracellular matrix microenvironment (ECM) by tumor cells are involved in cell adhesion, motility, intercellular communication and invasion. The tumor microenvironment is expansively modified and remodeled by proteases, resulting in important changes in both cell-cell and cell-ECM interactions and in the generation of new signals from the cell surface. Metalloproteinases belonging to the ADAMTS (a disintegrin and metalloprotease with thrombospondin motifs) family have been implicated in tissue remodeling events observed in cancer development, growth and progression. Here we investigated the subcellular localization of ADAMTS-1 in normal-*like* (MCF10-A) and tumoral (MCF7 and MDA-MB-231) human breast cells. ADAMTS-1 is a secreted protease found in the extracellular matrix. However, in this study we show for the first time that ADAMTS-1 is also present in the nuclei and nucleoli of the three mammary cell lines studied here. Our findings indicate that ADAMTS-1 has proteolytic functions in the nucleus through its interaction with aggrecan substrate.

## Introduction

The tumor microenvironment is expansively modified and remodeled by proteases, resulting in important changes in both cell-cell and cell-extracellular matrix (ECM) interactions and in the generation of new signals from the cell surface. Metalloproteinases belonging to the ADAMTS (a disintegrin and metalloprotease with thrombospondin motifs) family have been widely implicated in tissue remodeling events observed in cancer development, growth and progression [1].

The ADAMTS proteinases belong to a family of metalloproteinases that have extracellular matrix processing, organogenesis, and hemostasis functions. They are involved in remodeling of the extracellular matrix in physiological processes as well as in pathological states, including cancer [1]. ADAMTS were first characterized for their ability to cleave the Glu^373^-Ala^374^ bond in the interglobular domain of aggrecan [2, 3]. Several ADAMTS aggrecanases have been identified, among them aggrecanase-1 (ADAMTS-4) and aggrecanase-2 (ADAMTS-5). ADAMTS-4 and ADAMTS-5 are multi-domain metalloproteases secreted into the extracellular space. They both have a catalytic metalloprotease domain and a series of other ancillary domains regulating activity and substrate specificity [4].

A total of 19 ADAMTS proteases have been identified in humans. They share homology in the catalytic ADAM-metalloprotease and disintegrin domains, but differ in the variable numbers of thrombospondin-like motifs and other carboxyl-terminal domains that are associated with ECM interaction [5, 6]. ADAMTSs are not membrane-anchored proteinases, but after being secreted they do attach to the extracellular matrix [7, 8]. ADAMTS members act on a variety of ECM substrates but mostly on proteoglycans such as aggrecan [6], a major structural component of cartilage [9, 10].

ADAMTS-1 was initially described as a mediator of inflammation, but its activity has since become appreciated in organogenesis, blood/lymph vessel formation, ovarian folliculogenesis and ovulation. Numerous studies report changes in ADAMTS-1 mRNA and protein levels in tumor progression in the prostate, liver and mammary gland [1]. ADAMTS-1 was found to be spatiotemporally expressed in the human endometrium during the menstrual cycle and pregnancy, with its accumulation being associated with the onset and progression of decidualization *in vivo* [11]. Our group observed variable levels of ADAMTS-1 mRNA expression but lower levels of ADAMTS-1 protein expression in human breast cancers as compared to normal tissue, with a striking decrease observed in high-malignancy (triple-negative cases). Furthermore, the decrease was observed specially on tumor stroma [12].

ADAMTS-1 has been detected in a variety of carcinomas [13], and an imbalance of ADAMTS-1 expression is associated with several tumors. However, there are conflicting findings where both under expression and overexpression of this proteinase are found in primary tumors [12, 14]. ADAMTS-1 can cleave aggrecan found in the extracellular matrix and can also degrade versican [15, 16].

In this study, we analyzed ADAMTS-1, ADAMTS-4 and ADAMTS-5 localization by immunofluorescence. We also analyzed subcellular fractionation by Western blot in order to investigate their localization among cellular compartments in normal-like (MCF-10A) and tumoral human breast cells (MCF-7 and MDA-MB-231). Finally, we detected aggrecan in the nuclei by immunofluorescence and investigated the proteolytic role of the nuclear fraction, which might be related to the presence of ADAMTS-1.

## Material and Methods

### Cell lines and experimental culture conditions

MCF-10A cells were cultured in Dulbecco’s modified Eagle’s medium-F12 (DMEM-F12, Sigma) supplemented with 5% fetal bovine serum (FBS; Cultilab, Campinas, São Paulo, Brazil), 20 ng/ml epidermal growth factor (EGF), 10 ug/ml insulin, 0.5 ug/ml hydrocortisone, and 100 ng/ml cholera toxin. MCF-7 and MDA-MB-231 cells were cultured in Dulbecco’s modified Eagle’s medium (DMEM, Sigma) supplemented with 10% fetal bovine serum (FBS). HT1080 cells were culture in Eagle’s Minimum Essential Medium (MEM) supplemented with 10% fetal bovine serum (FBS). The cells were maintained in 75-ml cm^2^ flasks in a humidified atmosphere of 5% CO_2_ at 37°C.

### Transfection

MDA-MB-231 cells were transfected with commercially available siRNA targeting ADAMTS-1 (Santa Cruz Biotechnology Inc., Santa Cruz, CA, USA), according to the manufacturer’s instructions. One day prior to transfection, subconfluent MDA-MB-231 cells were cultured in DMEM supplemented with 10% FBS without antibiotic-antimycotic solution. The cells were incubated with a complex formed by the siRNA (50 nM), transfection reagent (Lipofectamine 2000, Invitrogen) and transfection medium (Opti-MEM I, Invitrogen) for 72 h at 37°C. Cells transfected with scrambled siRNA served as controls.

### Immunohistochemical analysis

Tissue microarray slides from normal human and breast cancer samples were obtained from Imgenex (San Diego, CA; IMH-364). 4-μm sections were analyzed. For antigen retrieval, 10 mM of citrate buffer (pH 6.0) with 0.05% Tween 20 was applied for 30 minutes at 100°C. Anti-ADAMTS-1 from Abcam (ab28284) was used (1:1000) overnight. EnVision method (EnVision; Dako Corp., Carpinteria, CA, USA) was used as detection system. Diaminobenzidine served as the chromogen and nuclei were counterstained by Mayer’s hematoxylin.

### Western blot

Western blots were carried out to compare ADAMTS-1, ADAMTS-4 and ADAMTS-5 levels in MCF-10A, MCF-7 and MDA-MB-231 cytoplasmic/nuclear fractions.

Nuclear–cytoplasmic fractionation was carried out using the NE-PER Nuclear and Cytoplasmic Extraction Reagents kit (Thermo Fisher Scientific) according to the manufacturer’s protocol, and then quantified (BCA kit, Pierce). Samples were resuspended in Laemmli buffer containing 62.5 mM Tris-HCl pH 6.8, 2% sodium dodecyl sulphate (SDS), 10% glycerol, 5% mercaptoethanol and 0.001% bromophenol blue. A total of 30 μg of the cellular proteins were electrophoresed on a 10% polyacrylamide gel, transferred to a Hybond ECL nitrocellulose membrane (Amersham) and blocked in Tris-buffered saline buffer (1X TBS) with 5% non-fat milk overnight at 4°C. Following one wash in TBS with 0.05% Tween 20 (TBST), the membranes were probed with antibodies against ADAMTS-1 (1:1000, Millipore MAB 1810), ADAMTS-4 (1: 1000, Abcam Ab84792), ADAMTS-5 (1:100, Abcam Ab39202), GAPDH (1:2000, Abcam Ab9484), histone (1:4000, Millipore 05–457), aggrecan (1:500, Sigma, SAB4500662, and Abcam 3778) and β-actin (1:2000, Sigma). The Clarity Western ECL Substrate (Bio-Rad) was used to detect proteins on the membrane according to the manufacturer’s protocol.

### Immunofluorescence

Cells grown on glass coverslips were fixed in 4% paraformaldehyde in phosphate-buffered saline (PBS) 1X for 10 min, rinsed and permeabilized with 0.5% Triton X-100 (Sigma) in PBS for 10 min, rinsed and blocked with normal goat serum (10%) for 1 hour. Cells were incubated with primary antibodies against ADAMTS-1 amino-terminal end (Abcam 28284 and Mab1810 Millipore), ADAMTS-4 C-terminal (Abcam Ab84792), and ADAMTS-5 C-terminal (Abcam Ab39202). All antibodies were rabbit polyclonal and the incubation was carried out for 1 hour at room temperature. Primary antibodies were revealed by anti-rabbit Alexa 568 (Invitrogen). Cells were further stained to F-actin by Alexa Fluor 488 phalloidin (Invitrogen). Samples were mounted in Pro Long with DAPI (Invitrogen).

To investigate co-localization of nucleophosmin and ADAMTS-1, we used anti-nucleophosmin primary monoclonal antibody (Sigma B0556). For aggrecan localization, we used anti-aggrecan primary monoclonal antibody (Abcam 3778). All antibodies were incubated for 1 hour at room temperature. Irrelevant rabbit IgG antibody was used as negative controls.

### Microscopy and image analysis

Samples subjected to immunofluorescence were analyzed by widefield fluorescence microscopy (Axiophot, Carl Zeiss, Oberkochen, Germany). Images (40X and 100X magnifications) were acquired by a digital CCD monochromatic camera (CoolSnap HQ2, Photometrics Inc., Tucson, AZ, USA). To assess nuclear extensions through the fluorescent substrate, at list ten Z sections per sample field were obtained using a piezoelectric device (PIFOC, Physik Instrumente, Germany) coupled to the objective. The microscope and all devices were controlled by Metamorph Premier 7.6 software (Molecular Devices, Sunnyvale, CA, USA).

A total of 10 random cells were imaged per experimental group. Orthogonal projections and image restoration through deconvolution algorithms were carried out by Volocity software (PerkinElmer, Waltham, MA, USA).

Measurement of colocalization areas and particles analysis was determined using ImageJ public domain software (http://rsb.info.nih.gov/ij/). Colocalization analysis was carried out by the Linescan tool (Metamorph Premier 7.6 software), and the Image J plugins JaCop, Colocalization colormap and Colocalization threshold.

To evaluate nucleolus ADAMTS-1 staining, red channel was analyzed; a threshold area was determined and then measured. Control and siRNA groups nucleolus area were compared.

### Aggrecan digestion and deglycosylation

Nuclear protein digestion assays were carried out in 100 ml of 50 mM Tris-HCl buffer (pH 7.5), containing 100 mM NaCl and 10 mM CaCl2. Aggrecan from bovine articular cartilage (500 nM) was incubated with 30 μg of nuclear fraction at 37°C overnight. Reactions were stopped by 0.5 M EDTA and samples were then deglycosylated.

For further fragment analysis by Western blot, aggrecan was enzymatically deglycosylated with chondroitinase ABC (0.01 units/10 μg of aggrecan) for 1 h at 37°C in buffer containing 50 mM sodium acetate and 100 mM Tris–HCl (pH 6.5). After digestion, the aggrecan was precipitated with five volumes of acetone and reconstituted in 30 ml of Tris-glycine SDS sample buffer containing 2.5% b-mercaptoethanol and heated for 3 min at 100°C and subjected to immunoblot analysis.

### Statistical analysis

Statistical analysis was performed using one-way ANOVA with the Graph Pad Prism 5 software (Graph Pad Software, Inc., San Diego, CA, USA).

## Results

### ADAMTS-1 is present in the nucleus

**Fig 1:**
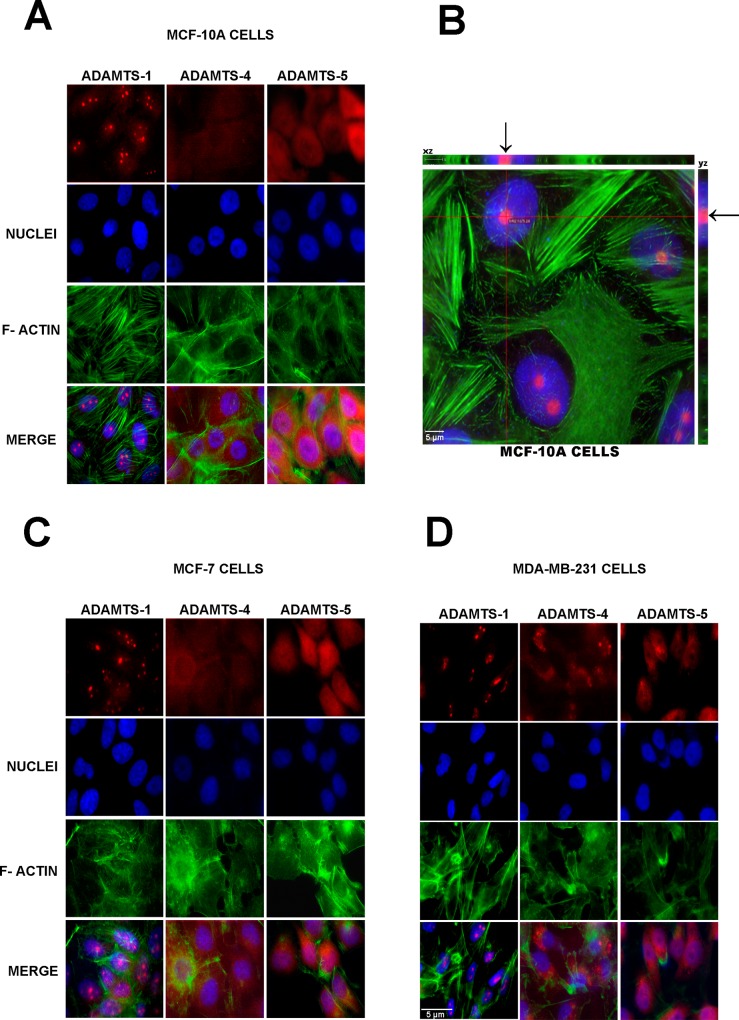
ADAMTS-1 is found predominantly in the nucleus. Immunofluorescence showing ADAMTS-1 localized in the nucleus, ADAMTS-4 and ADAMTS-5 are observed in cytoplasm in MCF-10A (A), MCF-7 (C) and MDA-MB-231 (D) cell lines. Colocalization of ADAMTS-1 and nucleus is evident in orthogonal projections (B, black arrows). Red lines in B indicate points of XY image projected to generate orthogonal planes XZ and YZ. ADAMTS-1, ADAMTS-4 and ADAMTS-5 (red), F-actin (green) and nuclei (blue). Scale bar: 5 μm.

The subcellular localization of ADAMTS-1, ADAMTS-4 and ADAMTS-5 was first determined using immunofluorescence. ADAMTS-1 was present primarily in the nucleus and with a relatively homogeneous distribution in MCF-10A (Fig 1A and 1B), MCF-7 (Fig 1C), and MDA-MB-231 (Fig 1D) cells. The same localization pattern was observed when the cells were stained with another commercially available mouse monoclonal antibody against ADAMTS-1, MAB 1810 from Millipore (S1A Fig). The negative control using irrelevant IgG is shown in S1B Fig. In HT1080, a cell line derived from fibrosarcoma, ADAMTS-1 is present mainly in the cytoplasm (S1C Fig).

Orthogonal projections XZ and YZ (arrows in Fig 1B) clearly demonstrate ADAMTS-1 inside the cells nuclei and colocalized with nucleoli.

Within the nucleus, ADAMTS-1 staining was round, forming well-defined structures. In contrast, ADAMTS-4 and ADAMTS-5 were not present in the nuclear compartment but rather distributed throughout the cytoplasm (Fig 1).

We observed ADAMTS-1 in the nuclei of all cell lines studied. To confirm ADAMTS-1 localization in the nuclei, we used immunofluorescence to address co-localization of ADAMTS-1 with nucleophosmin (a nucleolar protein).

**Fig 2:**
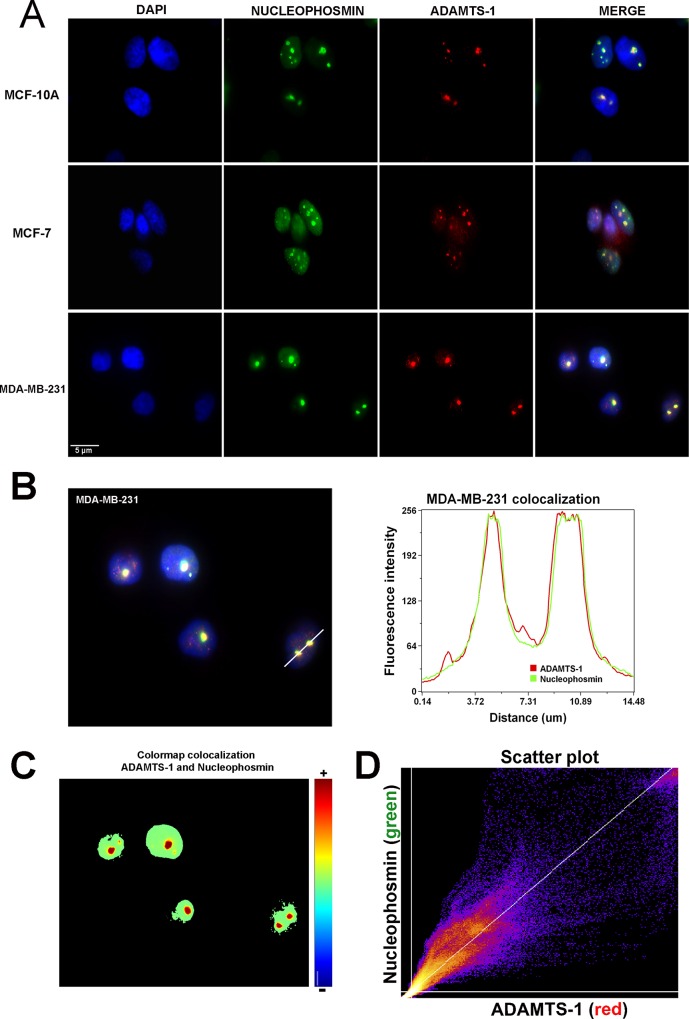
ADAMTS-1 is located in nucleolus. Immunofluorescence shows co-localization of ADAMTS-1 and nucleophosmin in MCF-10A, MCF-7 and MDA-MB-231 cell lines (A). MDA-MB-231 image used for colocalization analysis (B), with the white line used to generate the linescan plot (C). Linescan profiles display a clear overlap between ADAMTS-1 (Red) and nucleophosmin (Green). The colocalization color map is illustrated in C, where hot colors represent positive correlation (colocalization), and cold colors represent exclusion. Scatter plot (D) of ADAMTS-1 (red, x-axis) and nucleophosmin (green, y-axis) with the linear regression line. Points of scatterplot cluster around regression line. ADAMTS-1 (red), nucleophosmin (green) and nuclei (blue). Scale bar, 5 μm.

Immunofluorescence showed colocalization of ADAMTS-1 and nucleophosmin in all cell lines (Fig 2A). Furthermore, we carried out colocalization analysis in MDA-MB-231 cells (Fig 2B). The graphic in Fig 2B shows a perfect overlap between ADAMTS-1 (red channel) and nucleophosmin (green channel). Colormap (Fig 2C) and scatterplot (Fig 2D) confirm colocalization of ADAMTS-1 and nucleophosmin. Ten cells were analyzed and mean Pearson's correlation coefficient was R = 0.9438 (standard error = 0.002263).

To confirm the nuclear localization of ADAMTS-1 previously observed by immunofluorescence staining (Fig 1), we obtained nuclear and cytoplasmic fractions of the three studied cell lines and evaluated the expression of ADAMTS-1, ADAMTS-4 and ADAMTS-5 by immunoblotting.

**Fig 3:**
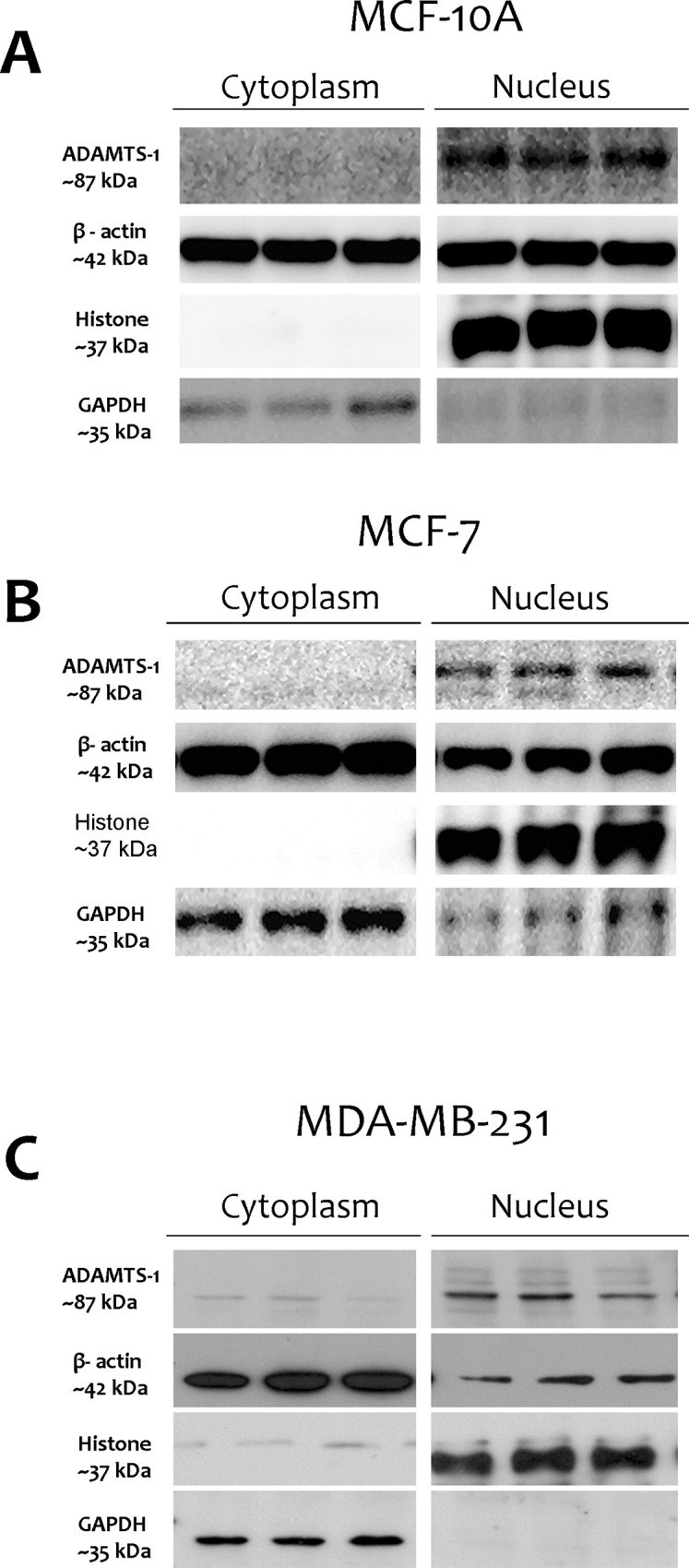
ADAMTS-1 protein is predominantly in the cell nucleus. Western blot containing cytoplasmic and nuclear fractions from MCF-10A, MCF-7, and MDA-MB-231 cells, probed for ADAMTS-1, β-actin, histone and GAPDH.

**Fig 4:**
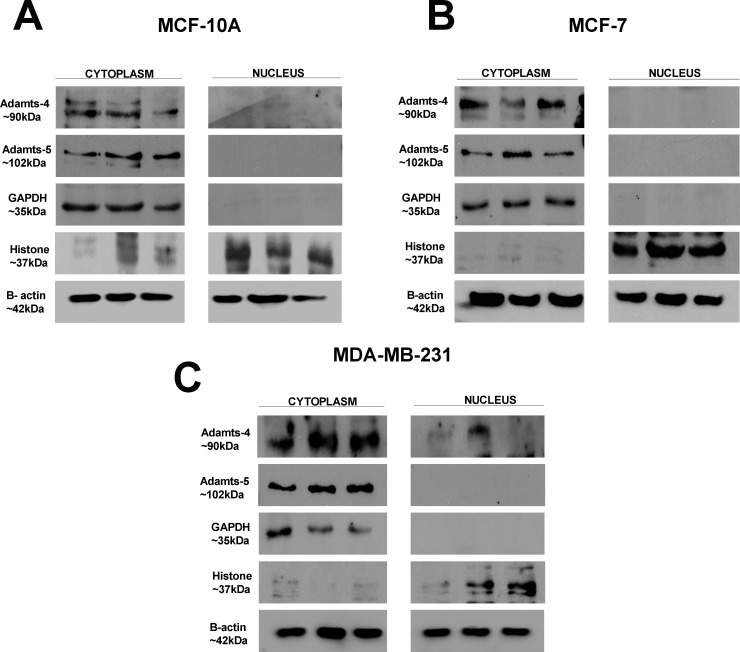
ADAMTS-4 and ADAMTS-5 are found in the cytoplasm. Western blot containing cytoplasmic and nuclear fractions from MCF-10A (A), MCF-7 (B) and MDA-MB-231 (C) cells, probed for ADAMTS-4, ADAMTS-5, β-actin, histone and GAPDH.

ADAMTS-1 was not found in cytoplasmic fractions from either MCF-10A or MCF-7 cells (Fig 3A and 3B). On the other hand, this protease was found in the nuclear fractions from both MCF-10A and MCF-7 cells. In MDA-MB-231 cells, ADAMTS-1 was observed in both the nuclear and cytoplasmic fractions, but predominantly in the nuclear fraction (Fig 3C). Western blots for ADAMTS-1 confirmed its presence in all nuclear extracts, predominantly in the 87 kDa form. ADAMTS-4 and ADAMTS-5 signals were predominant in the cytoplasmic fractions of all cell lines studied, compared to the signal observed in the nuclear fraction (Fig 4). ADAMTS-4 and ADAMTS-5 were not found in the nuclear fractions of either MCF-10A or MCF-7 cells (Fig 4A and 4B). In MDA-MB-231 cells, the nuclear fraction exhibited only a weak ADAMTS-4 signal (Fig 4C).

### Aggrecan cleavage by the nuclear fraction

Aggrecan is a substrate recognized by the aggrecanases ADAMTS-1, ADAMTS-4 and ADAMTS-5.

**Fig 5:**
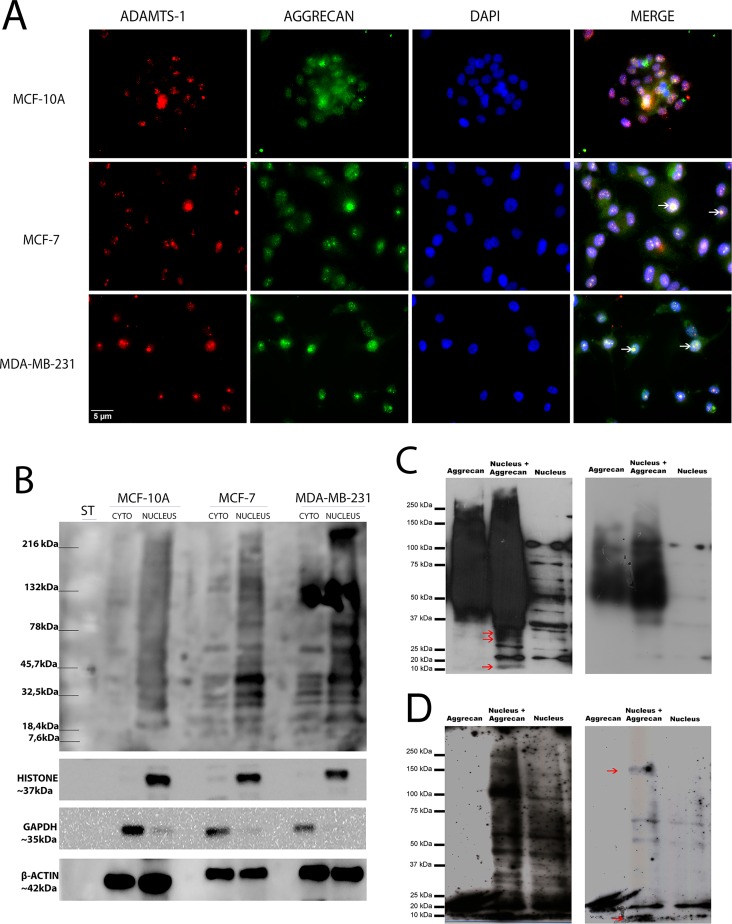
ADAMTS-1 and Aggrecan are both in the nucleus. (A) Colocalization of ADAMTS-1 and aggrecan. ADAMTS-1 (red), Aggrecan (green) and Nuclei (blue). Aggrecan is found in nuclei and cytoplasm and is colocalized with ADAMTS-1 in cell nucleolus (white arrows) in MCF7 and MDA-MB-231. Scale bar: 5 μm. (B) Western blot containing cytoplasmic and nuclear fractions from MCF-10A, MCF-7, and MDA-MB-231 cells, probed for Aggrecan, β-actin, histone and GAPDH. (C-D) Aggrecan Western blot after digestion. Lane 1: Aggrecan from bovine articular cartilage (500 nM); Lane 2: Aggrecan from bovine articular cartilage (500 nM) incubated with 30 μg of MCF-7 nuclear fraction; Lane 3: 30 μg of MCF-7 nuclear fraction. All samples were subjected to digestion (overnight at 37°C) and a deglycosylation assay (1 hour at 37°C). Red arrows indicate proteolytic product from nuclear proteases. The same membrane was probed with two different commercial antibodies. (C) High and low exposure using anti-aggrecan from Sigma (SAB 4500662). (D) High and low exposure using anti-aggrecan from Abcam (#3778).

To confirm whether ADAMTS-1 in the cell nucleus would function as a protease, we analyzed the nuclear presence of aggrecan, an ADAMTS-1 substrate. We found aggrecan in the nucleus in the same compartment where we observed ADAMTS-1 in MCF-7 and MDA-MB-231 cells. Aggrecan was also found in the nuclei of MCF-10A cells, but not entirely colocalized with ADAMTS-1 (Fig 5A). Immunoblot showed aggrecan in nuclear fraction of all cell lines (Fig 5B), thus confirming immunofluorescence findings.

To investigate whether ADAMTS-1 would have a proteolytic role in this cellular compartment, we carried out aggrecan digestion and deglycolysation assays. We observed that proteases in the nuclear fraction play a proteolytic function in MCF-7 cells, as their presence generated aggrecan fragments not observed in the control (incubated alone) or in aggrecan samples deprived of the nuclear fraction. These observations support the idea that ADAMTS-1 has a proteolytic activity in the cell nucleus (Fig 5C and 5D, red arrows). We used two different antibodies that recognized different band sizes.

### ADAMTS-1 knockdown

**Fig 6:**
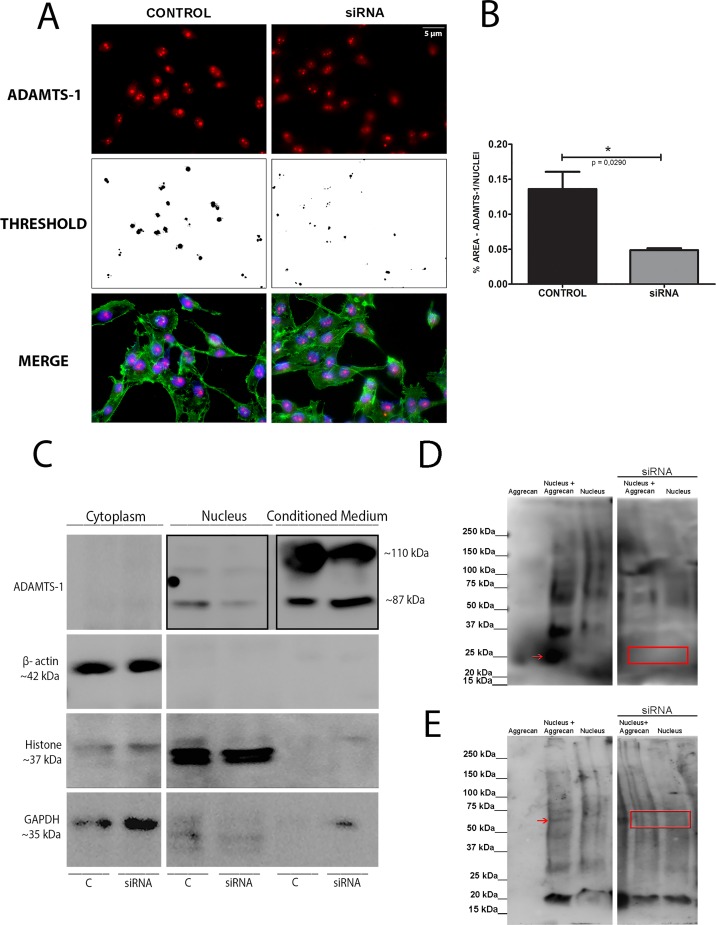
siRNA treatment decreases ADAMTS-1 nuclear pattern in MDA-MB-231 cells. (A) Immunofluorescence shows reduced area of ADAMTS-1 (red) at nuclei with siRNA treatment. (B) Quantification of ADAMTS-1 presence at nuclei was performed with Image J. Nuclear particle size is smaller with decreased ADAMTS-1 when compared to control. (C) Immunoblot shows the decrease of ADAMTS-1 at nuclear fraction and conditioned medium in cells treated by siRNA (boxed). (D-E) Aggrecan Western blot after digestion. Lane 1: Aggrecan from bovine articular cartilage (300 nM); Lane 2: Aggrecan from bovine articular cartilage (300 nM) incubated with 30 μg of MDA-MB-231 nuclear fraction; Lane 3: 30 μg of MDA-MB-231 nuclear fraction. Lane 4: Aggrecan from bovine articular cartilage (300 nM) incubated with 30 μg of MDA-MB-231 nuclear fraction (siRNA treated). Lane 5: 30 μg of MDA-MB-231 nuclear fraction (siRNA treated). All samples were subjected to digestion (overnight at 37°C) and a deglycosylation assay (1 hour at 37°C). Red arrows indicate proteolytic product from nuclear proteases. Boxed areas (red) indicate absence of proteolytic bands. The same membrane was probed with two different commercial antibodies. (D) Membrane probed with anti-aggrecan from Sigma (SAB 4500662). (E) Membrane probed with anti-aggrecan from Abcam (#3778).

To further evaluate nuclear localization of this protease, ADAMTS-1 was depleted by small interfering RNA (siRNA) in MDA-MB-231 cells. Cells showed reduced expression of ADAMTS-1 by immunofluorescence and further particle size analysis (Fig 6A and 6B), confirming depletion efficiency Immunoblot showed decreased ADAMTS-1 in nuclear fraction and conditioned medium (Fig 6C). Nuclear fractions from cells with reduced expression of ADAMTS-1 were incubated with aggrecan. In this situation no digested aggrecan fragments were observed (Fig 6D and 6E).

### ADAMTS-1 staining on tissue sections

**Fig 7:**
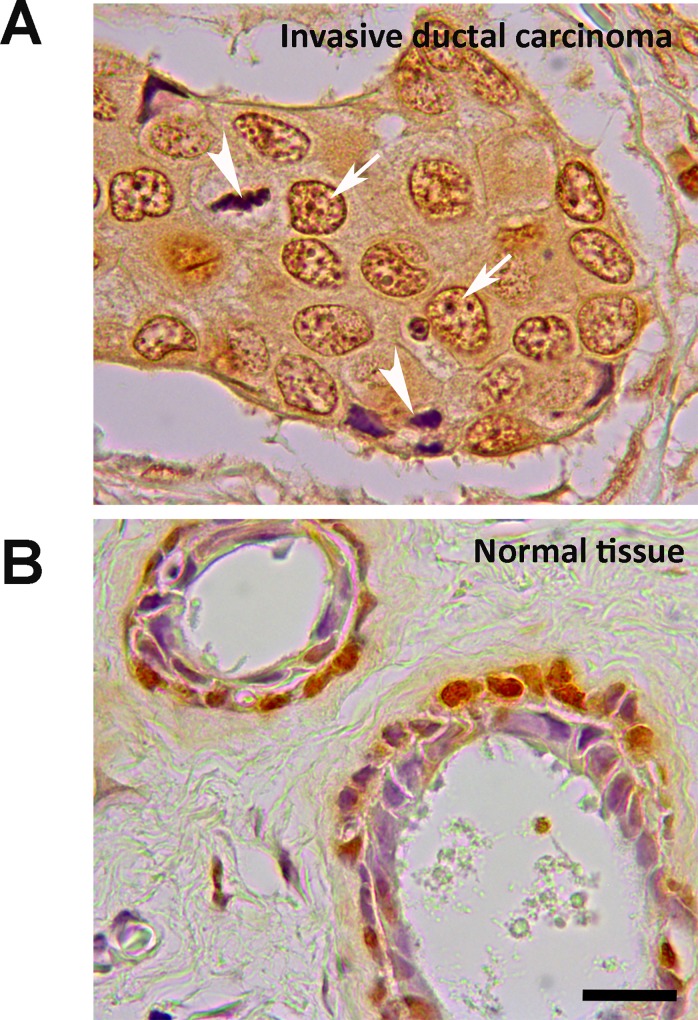
ADAMTS-1 is present on nuclei of invasive ductal carcinoma cells but not on luminal cells of normal breast tissue. (A) Immunohistochemistry shows ADAMTS-1 inside the nucleus of cancer cells (arrows pointed to dot-like structures). Cells under division or stromal-like cells do not present ADAMTS-1 (arrowheads). (B) In normal tissue ADAMTS-1 is present on nuclei and cytoplasm of basal cells but not on luminal cells. Scale bar: 50 μm.

We analyzed by immunohistochemistry the presence of ADAMTS-1 on tissue sections. ADAMTS-1 is present in nuclei of invasive ductal carcinoma cells as dot-like structures (Fig 7A). In normal breast tissue ADAMTS-1 was present in nuclei and cytoplasm of myoepithelial cells but not on luminal cells (Fig 7B).

## Discussion

In this study, we show that ADAMTS-1 is present in the nuclei and nucleoli of the three mammary cell lines studied here.

To our knowledge, this is the first time that a member of the ADAMTS family has been detected in the cell nucleus. We observed the presence of ADAMTS-1 in the nucleus by both immunofluorescence and immunoblotting methods. The antibody recognized the 87kDa band, corresponding to activated protease. We also observed co-localization of ADAMTS-1 and nucleophosmin, which is a nucleolar protein.

A number of proteases have been found in the nucleus, including members of the cysteine protease and cathepsin families. For instance, a previous study showed that MMP-2 (matrix metalloproteinase-2) is present in the nucleus of heart and liver cells, and was localized in the nuclei of cardiac myocytes [17]. In addition, studies have shown that MMP-3 and TIMP-1 (metallopeptidase inhibitor 1) are also present in the nucleus and that MMP-3 is involved with apoptosis [18]. Additionally, MMP-3 fragments were observed in the nucleus of several tumor cell lines [19]. The serine protease HtrA1 was also described in the nuclei of MCF-10A cells as well as breast tissues [20].

The functions of these proteases are not well understood, but may include chromatin remodeling, maintenance of nuclear matrix structure, apoptosis, and regulation of cellular proliferation [21]. The mechanism of action for nuclear ADAMTS-1 is still unclear, and a nuclear localization signals (NLS) has not yet been described for this protease. For example, a study demonstrates that the protein content of the nuclear matrix works similarly to the extracellular matrix, by anchoring many molecules. Thus, our finding, together with others, suggests this possible biological roles for metalloproteinases in the nucleus [17].

ADAMTS-1 is a secreted protein found in the extracellular matrix. The presence of ADAMTS-1 in the nucleus and nucleolus prompted us to investigate its function in this cellular compartment. We have also attempted to identify putative interactive molecules. Our data indicate that ADAMTS-1 can have a proteolytic function in the nucleus as shown by its interaction with aggrecan, an ADAMTS-1 substrate.

We previously described a decrease of ADAMTS-1 in triple negative tumors stroma [12]. Here we show the presence of ADAMTS-1 on tumor cell nuclei. Taken together, these results may suggest that this protease could be removed by endocytosis from the extracellular matrix and accumulated in the cell nuclei. Other groups already described the endocytosis of the Dentin Matrix Protein1, an extracellular matrix protein, and its accumulation inside cell nuclei [22]. A recent published paper describes a number of possibilities that explain the presence of extracellular matrix proteins inside the cytoplasm and nuclei. These explanation includes: alternative splicing of mRNA resulting in omission of secretory signal peptide, modulation of ER stress, ECM proteins can escape the endo/lysossomal system and enter the cytoplasm or nucleus, a number of ECM protein contain a nuclear localization and ECM proteins might go to cytosol using ER-associated degradation pathway [23]. Since we observed nuclear ADAMTS-1 on tissue tumor cells and on cells in culture, but not on normal breast luminal cells, further studies can explain if these pathways are altered in tumor cells compared to normal breast cells.

The identification of specific matrix and non-matrix substrates for MMPs and new knowledge gained about the cleavage of such substrates indicate that several MMPs are not limited to matrix substrates, which may explain their novel and unexpected nuclear localization [24]. A molecular and immunocytochemistry study carried out to assess expression of MMPs in normal breast and breast cancer cells showed that MMP-1 is predominantly found in the nuclei of tumor cells, with a small amount of cytoplasmic distribution [25], whereas no signal was observed in normal breast tissue [26]. Another study reported a novel transcription factor-like function of MMP-3 in the nuclei of chondrocytic cells in culture [27].

In this study, we have shown that ADAMTS-1 is found in the nucleus, which indicates that it may play a proteolytic function such as cleavage of nuclear aggrecan. Aggrecan is a well-known ADAMTS-1 substrate and here we demonstrate that aggrecan is also present in breast cells nuclei. Chen et al. also described that aggrecan domains can be accumulated in the cell nuclei [28]. In a previous work from our group we observed by immunohistochemistry the presence of aggrecan in the nuclei of ovarian tumors cells [29]. Our results showing that nuclear fractions from MCF-7 and MDA-MB-231 generated aggrecan fragments support this idea. Furthermore ADAMTS-1 is involved in this process, since nuclear fraction from cells with reduced expression of ADAMTS-1 failed to generate aggrecan fragments observed before.

We cannot rule out that the nuclear localization of MMP may have resulted from a faulty process that misdirected the protein to the nucleus. However, identification of a nuclear localization signal in some MMPs suggests a bona fide and alternative nuclear localization that may represent an important and yet unknown function of MMPs. In fact, there is increasing evidence that subspecialized and compartmentalized nuclear MMPs may participate in many physiological and pathological processes [24].

Future studies should be carried out to provide new insights into the transcriptional and translational control of MMP expression, to investigate its transport and trafficking, and to thoroughly identify new nuclear substrates for these enzymes. This information is crucial for a better understanding of the functions and roles of MMPs in general. The precise mechanism by which ADAMTS-1 recognizes and cleaves other players in the cell nucleus remains unclear, and would be the aim of future investigations.

## Conclusion

Our study is the first to show that ADAMTS-1 is present in the nuclei of breast cell lines, where its expression is more abundant than that observed in the cytoplasm. In addition, ADAMTS-1 may generate aggrecan fragments when in the nuclear compartment, suggesting that ADAMTS-1 has proteolytic activity within nuclei as well as in the extracellular matrix.

## Supporting Information

S1 Fig MAB 1810 (Millipore) also recognizes ADAMTS-1 in nuclei. (A) Immunofluorescence was performed in MCF-10A cells with another commercially available antibody against ADAMTS-1, and the same labeling pattern is observed. (B) Negative controls of immunofluorescence assays; Non-immune IgG (Red), F-actin (Green) and Nuclei (Blue). (C) Immunofluorescence assay of HT1080 cells showing ADAMTS-1 mainly in cytoplasm. Scale bar: 5 μm.
(TIF)
