# Supplementary figures and images for: ADAMTS-1 Is Found in the Nuclei of Normal and Tumoral Breast Cells
Source: PLoS One. 2016 Oct 20;11(10):e0165061. doi: 10.1371/journal.pone.0165061 (PMC5072708; doi:10.1371/journal.pone.0165061)

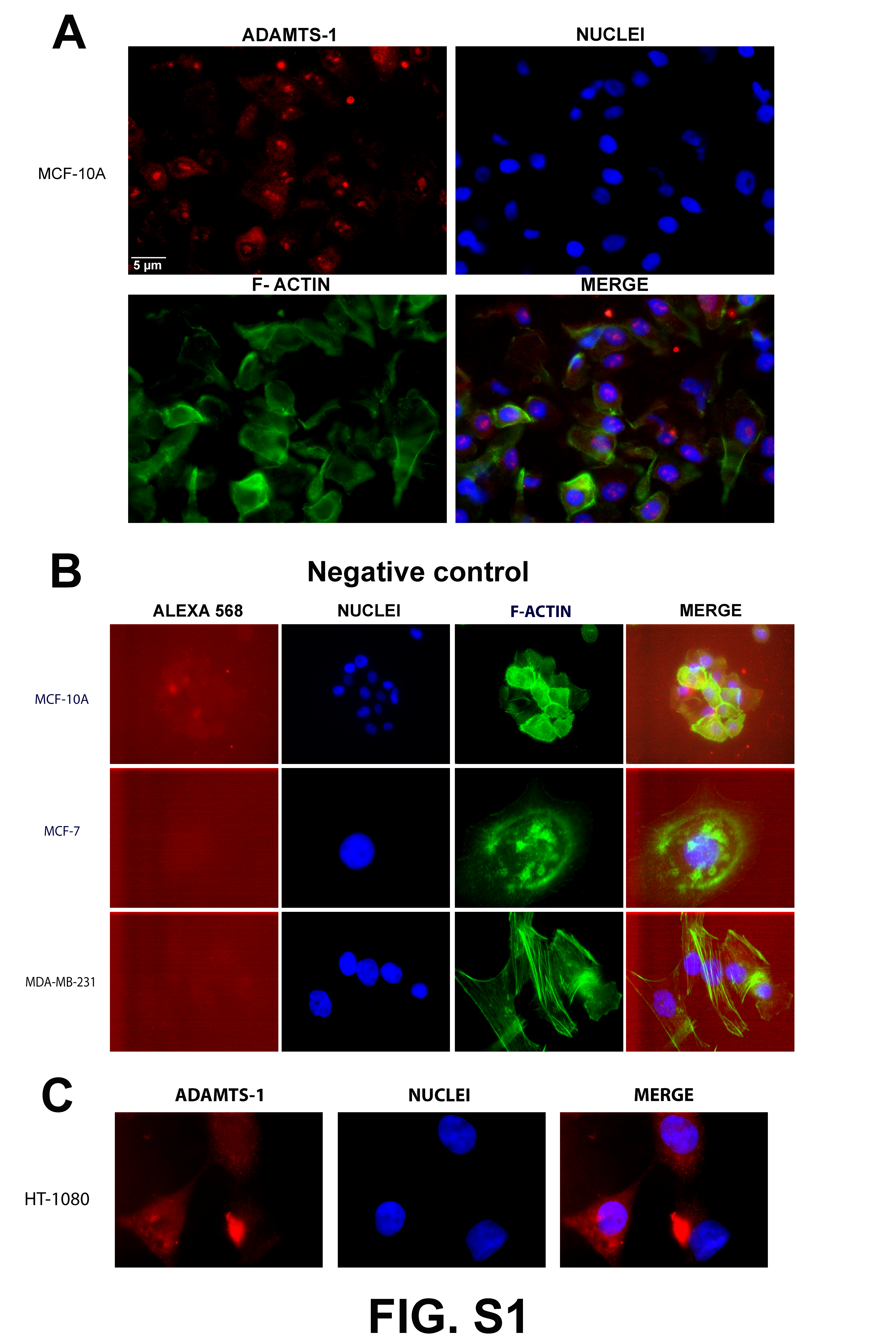

Supplement: S1 Fig — (A) Immunofluorescence was performed in MCF-10A cells with another commercially available antibody against ADAMTS-1, and the same labeling pattern is observed. (B) Negative controls of immunofluorescence assays; Non-immune IgG (Red), F-actin (Green) and Nuclei (Blue). (C) Immunofluorescence assay of HT1080 cells showing ADAMTS-1 mainly in cytoplasm. Scale bar: 5 μm. (TIF) [file pone.0165061.s001.tif]
